# Pedometer-determined physical activity among youth in the Tokyo Metropolitan area: a cross-sectional study

**DOI:** 10.1186/s12889-016-3775-5

**Published:** 2016-10-21

**Authors:** Noritoshi Fukushima, Shigeru Inoue, Yuki Hikihara, Hiroyuki Kikuchi, Hiroki Sato, Catrine Tudor-Locke, Shigeho Tanaka

**Affiliations:** 1 Department of Preventive Medicine and Public Health, Tokyo Medical University, 6-1-1 Shinjuku Shinjuku-ku, Tokyo, 160-8402 Japan; 2 Faculty of Engineering, Chiba Institute of Technology, 2-1-1 Shibazono, Narashino, Chiba 275-0023 Japan; 3 Department of Kinesiology, School of Public Health and Health Sciences, University of Massachusetts Amherst, 111 Totman Building University of Massachusetts Amherst 30 Eastman Lane, Amherst, MA 01003-9258 USA; 4 Department of Nutritional Science, National Institute of Health and Nutrition, National Institutes of Biomedical Innovation, Health and Nutrition, 1-23-1 Toyama, Shinjuku-ku, 162-8636 Tokyo, Japan

**Keywords:** Survey, Steps, Children, Adolescents, Cross-sectional study, Descriptive epidemiology

## Abstract

**Background:**

Providing large-scale descriptive data of objectively measured physical activity in youth is informative for practitioners, epidemiologists, and researchers. The purpose of this study was to present the pedometer-determined physical activity among Japanese youth using the Tokyo Metropolitan Survey of Physical Fitness, Physical Activity and Lifestyle 2011.

**Methods:**

This study used a school-based survey. The Tokyo Metropolitan Board of Education originally collected pedometer-determined steps per day in the fall of 2011. Data were collected from 15,471 youth aged 6 to 18 years living in Tokyo. Participants were asked to wear pedometers for 14 consecutive days, and daily steps logged in the final 7 days were selected for this analysis.

**Results:**

At the primary and junior high school levels, boys (12,483 and 9476, respectively) had a significantly higher mean number of steps per day than did girls (10,053 and 8408, respectively). There was no significant difference in the mean number of steps per day between the sexes at the high school level. Mean steps per day decreased consistently with age and grade level; the lowest overall steps per day was observed in the last year of junior high school, although there was a slight increase in the subsequent year, the first year of high school.

**Conclusions:**

This study demonstrates a trend toward reduced physical activity with age in Japanese youth and a substantial difference in the number of steps per day between boys and girls in Tokyo. The age-related reduction in steps per day was greater in boys because they attained a higher peak value prior to this reduction, and sex-related differences in the step count disappeared in high school students.

## Background

Lack of physical activity (PA) in childhood and adolescence is associated with adverse health problems such as obesity and increased cardiovascular and diabetes risk [1, 2]. Childhood PA patterns often extend into adulthood; insufficient PA during this developmental period is therefore a great public health threat [3]. To improve health outcomes, the World Health Organization recommends that children and adolescents (hereafter collectively termed “youth”) aged 5 to 17 years participate in a daily minimum of 60 min of moderate to vigorous PA [4]. Despite these recommendations, physical activity levels among youth remain low worldwide [5, 6].

Previous studies that evaluated PA levels primarily used standardized self-report questionnaires [2, 5, 6]. Although self-reporting is reasonable for large-scale epidemiological investigations, it may be less appropriate for measuring PA in children and adolescents. For example, recall bias may affect the accuracy of child data more than adult data [7]. Additionally, children may be unable to accurately summarize the sporadic and complex nature of their PA when responding to questions about habitual behavior [8, 9]. Further, cross-national comparisons of self-reported PA are affected by language and cultural differences [10–12]. Collectively, these concerns indicate a substantial need for objectively measured youth PA [13].

Pedometers and accelerometers are commonly used to objectively measure PA and are increasingly used as research tools. It has also been reported that these devices are valid and feasible in assessing PA in youth [14]. Moreover, pedometers are more cost-effective than accelerometers; the number of steps per day provides simple and practical information about PA volume for researchers, practitioners, and the lay public. There are a few examples of national surveys of young people’s objectively determined steps per day: the Canadian Physical Activity Levels among Youth (CANPLAY) survey used pedometers [15, 16], the U.S. National Health and Nutrition Examination Survey used accelerometers [17], and the European Youth Heart Study also used accelerometers [18]. Still, evidence regarding objectively measured PA among Asian youth are quite limited [5].

We aimed to examine descriptive epidemiological data for children’s and adolescents’ pedometer-determined PA levels using the Tokyo Metropolitan Survey of Physical Fitness, Physical Activity and Lifestyle 2011. Specifically, we provide descriptive epidemiological data on the number of steps per day, stratified by sex and grade level.

## Methods

**Fig. 1:**
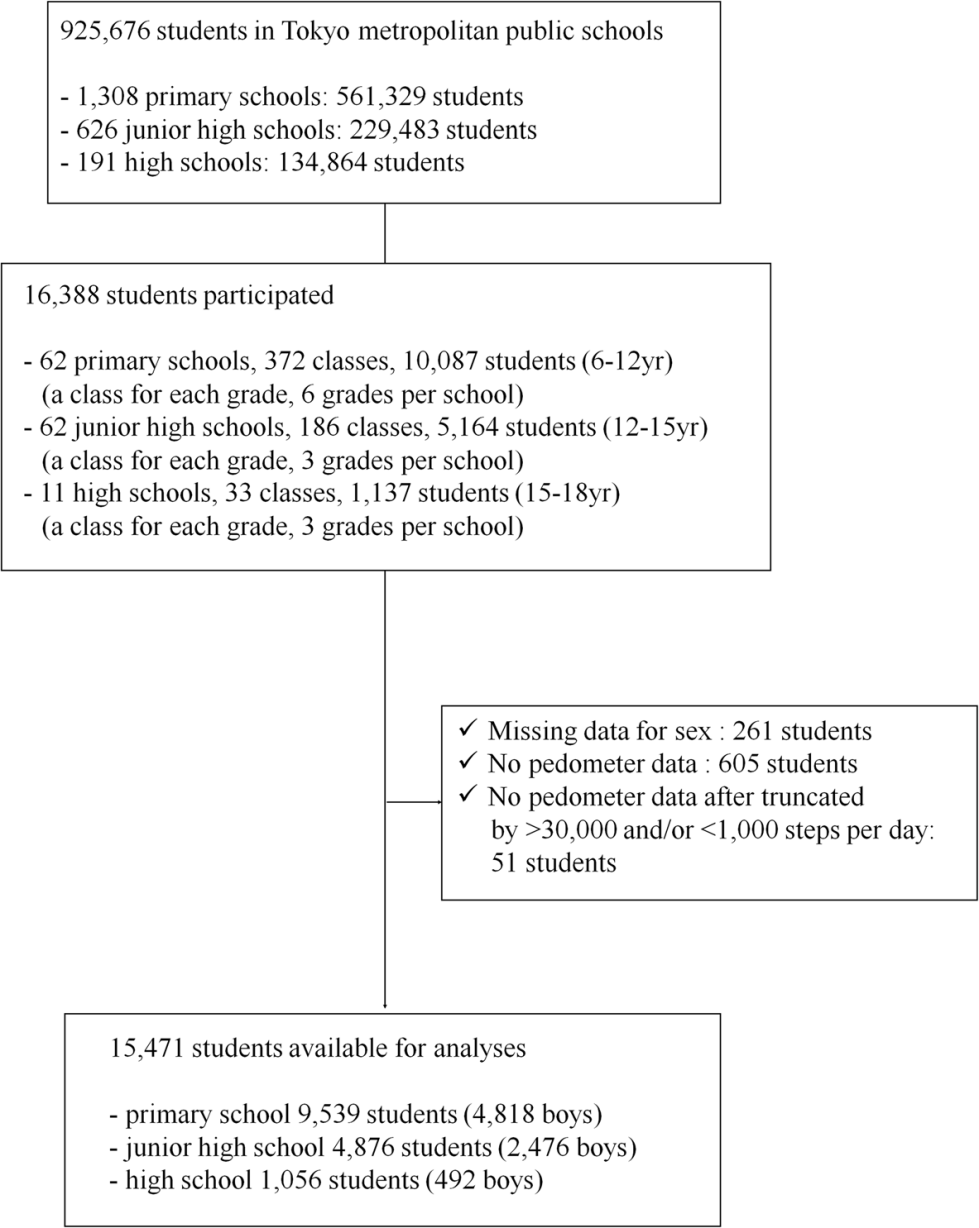
Participant sampling flow chart and strategy for data assessment

A schematic depicting the sampling and data assessment methodology used in this study is shown in Fig. 1.

### Data source

The Tokyo Metropolitan Board of Education (TMBE) performed a cross-sectional survey to investigate PA in youth living in Tokyo by examining the number of pedometer-recorded steps per day during the 2011 fall academic term. The TMBE authority and the Tokyo Metropolitan Government approved the secondary use of these data for research purposes, and all provided data were stripped of personal identifiers.

### Participants and data collection

Primary school and junior high school are compulsory in Japan. Children are admitted to primary school at 6 years of age. They spend 6 years in primary school, followed by 3 years in junior high school. After graduation from public junior high school in 2011, 97.6 % of students in Tokyo attended high school for 3 years [19]. In Tokyo in June 2011, there were 561,329 students registered in 1308 public primary schools, 229,483 students in 626 public junior high schools, and 134,864 students in 191 public high schools. Geographically, the Tokyo metropolitan comprises 2 areas and 2 islands containing 62 municipalities: 23 Ku-Area (23 wards), Tama-Area (26 cities, 3 towns, and 1 village), and Izu and Ogasawara Islands (2 towns and 7 villages). This is a secondary analysis of a TMBE survey that did not employ random sampling. Instead each of the 62 municipalities of Tokyo were asked, at their own discretion to designate one public primary school and one public junior high school from their jurisdiction for targeted measurement. Thus, the data came from 62 primary schools and 62 junior high schools throughout Tokyo. One class per grade in each of these schools participated in this survey. The TMBE sampled high school students from the 11 school districts of the Tokyo metropolitan.

Data were collected from 10,087 students from 62 primary schools (372 classes), 5164 students from 62 junior high schools (186 classes), and 1137 students from 11 public high schools (33 classes). Participants were aged 6 to 18 years. Each school held an orientation meeting for this survey for participants and their parents or guardians in August 2011. Data were collected using pedometers and questionnaires during the fall term of 2011 (September to November).

### Pedometer-determined PA

TMBE members chose the pedometer used in the survey (EX-200; Yamasa Co., Ltd., Tokyo, Japan; approximately $US 23); Yamasa is the Japanese generic name for Yamax, and this brand has been commonly used among PA researchers [20, 21]. In addition, our previous study reported acceptable comparability of the EX-200 compared with the SW-200 (Yamax Co., Ltd., Tokyo, Japan), the Kenz Lifecorder (Suzuken Corp., Nagoya, Japan), and the Active style Pro HJA-350IT (Omron Healthcare, Kyoto, Japan) among Japanese children [22]. Pedometers were placed in participants’ pockets for data collection in the present study. The EX-200 can store up to 7 days of memory data, and students recorded their step counts daily using the memory function at school under the guidance of trained teachers. Participants were asked to wear an unsealed pedometer during waking hours for 14 consecutive days; they were allowed to remove the device for water-based activities and while engaging in full-contact sports (e.g., judo). The number of steps per day during the first 7 days of monitoring was not recorded in accordance with the original TMBE survey protocol. Therefore, the data for the remaining 7 days were used in these analyses.

### Data treatment and statistical analyses

Step data were treated similarly to those of the CANPLAY survey [23] to enable comparison of both sets of results. Because a single day of pedometer data can be used to accurately estimate PA levels for surveillance purposes, participants aged 6 to 18 years with at least 1 valid day of pedometer data were included in this analysis [23, 24]. Records of <1,000 or >30,000 steps per day were considered outliers and excluded from further analyses [15, 20, 23]. Valid days were thus defined as any day with recorded data between these two thresholds. Descriptive data (means, 95 % confidence intervals [CI]) for the number of steps per day were calculated based on the number of valid days for each grade level and sex (combined and separately). Ranges and percentile values were calculated for each grade level by sex. In Japan, an evidence-based recommendation for steps per day for youth aged 6 to 18 years has not yet been established. Therefore, we used criteria applied in past studies to describe the proportion of participants taking ≥10,000, ≥12,000, and 15,000 steps per day [15, 25, 26]. Specifically, these criteria were a separate body mass index-referenced criteria for boys and girls (15,000 and 12,000 steps/day, respectively) [15, 26] and a step count related to 60 min of moderate to vigorous PA for adolescent boys and girls (10,000 steps per day) [25]. Moreover, 15,000 steps per day is the target recommended by the Tokyo Metropolitan Government for boys and girls aged 6 to 18 years [27] and <7000 steps per day is a potential candidate for the lower threshold in children, which indicates a sedentary lifestyle [28]. Finally, an accumulated <5000 steps per day (originally considered to indicate a sedentary lifestyle for adults) was used as an alternative marker of a sedentary lifestyle [28]. Student’s *t*-test was used to test for sex differences, stratified for each grade level. Cohen’s *d* effect size index was used to assess the magnitude of intergroup differences and statistical significance [29]. All statistical procedures and calculations of *p*-values were conducted using two-tailed t-tests. Differences were considered statistically significant at *p* < 0.05. Statistical analyses were performed using IBM SPSS software, version 21.0 (IBM, Armonk, NY, USA).

## Results

A total of 16,388 students participated in this survey. We excluded 261 students whose sex was unspecified in the data set, 605 students without any pedometer data recorded during the final 7 days of the monitoring period, and 51 students with no pedometer data after truncation to <1000 or >30,000 steps per day as data outliers. Thus, step-defined PA was successfully measured for 15,471 students, each with at least 1 valid day of data. Overall, 3.1 % of boys and 1.6 % of girls had only 1 valid day of pedometer data; 86.2 % of boys and 90.7 % of girls had ≥4 valid days of pedometer data (Table 1).

**Table 1:** Distribution of participants by valid days of pedometer wear, grade, and sex in Tokyo in 2011

					Number of students per valid days of pedometer wear													
			Total		1 day		2 days		3 days		4 days		5 days		6 days		7 days	
	Grade	Age	*n*	Pct	*n*	Pct	*n*	Pct	*n*	Pct	*n*	Pct	*n*	Pct	*n*	Pct	*n*	Pct
Boys																		
Primary school	All grades		7786	100	245	3.1	344	4.4	490	6.3	830	10.7	1220	15.7	1572	20.2	3085	39.6
	1	6–7	802	100	22	2.7	38	4.7	48	6.0	74	9.2	117	14.6	182	22.7	321	40.0
	2	7–8	772	100	30	3.9	37	4.8	55	7.1	76	9.8	115	14.9	179	23.2	280	36.3
	3	8–9	768	100	33	4.3	46	6.0	52	6.8	92	12.0	106	13.8	168	21.9	271	35.3
	4	9–10	828	100	33	4.0	33	4.0	47	5.7	84	10.1	132	15.9	173	20.9	326	39.4
	5	10–11	795	100	22	2.8	35	4.4	41	5.2	77	9.7	136	17.1	155	19.5	329	41.4
	6	11–12	853	100	27	3.2	29	3.4	45	5.3	77	9.0	123	14.4	181	21.2	371	43.5
Junior high school	1	12–13	877	100	34	3.9	40	4.6	70	8.0	119	13.6	151	17.2	174	19.8	289	33.0
	2	13–14	783	100	16	2.0	33	4.2	59	7.5	106	13.5	131	16.7	146	18.6	292	37.3
	3	14–15	816	100	23	2.8	38	4.7	52	6.4	79	9.7	128	15.7	138	16.9	358	43.9
High school	1	15–16	177	100	3	1.7	9	5.1	13	7.3	15	8.5	34	19.2	16	9.0	87	49.2
	2	16–17	161	100	0	0.0	4	2.5	3	1.9	21	13.0	21	13.0	30	18.5	82	50.9
	3	17–18	154	100	2	1.3	2	1.3	5	3.2	10	6.5	26	16.9	30	19.5	79	51.3
Girls																		
Primary school	All grades		7685	100	124	1.6	232	3.0	357	4.6	627	8.2	949	12.3	1575	20.5	3821	49.7
	1	6–7	762	100	18	2.4	26	3.4	47	6.2	78	10.2	88	11.5	160	21.0	345	45.3
	2	7–8	776	100	12	1.5	31	4.0	37	4.8	83	10.7	92	11.9	144	18.6	377	48.6
	3	8–9	797	100	18	2.3	33	4.1	36	4.5	60	7.5	88	11.0	181	22.7	381	47.8
	4	9–10	787	100	12	1.5	22	2.8	39	5.0	68	8.6	97	12.3	162	20.6	387	49.2
	5	10–11	796	100	11	1.4	20	2.5	22	2.8	47	5.9	76	9.5	166	20.9	454	57.0
	6	11–12	803	100	10	1.2	14	1.7	34	4.2	50	6.2	91	11.3	146	18.2	458	57.0
Junior high school	1	12–13	838	100	12	1.4	24	2.9	53	6.3	73	8.7	134	16.0	184	22.0	358	42.7
	2	13–14	746	100	11	1.5	16	2.1	36	4.8	70	9.4	81	10.9	158	21.2	374	50.1
	3	14–15	816	100	13	1.6	31	3.8	33	4.0	58	7.1	108	13.2	166	20.3	407	49.9
High school	1	15–16	197	100	5	2.5	7	3.6	8	4.1	20	10.2	33	16.8	39	19.8	85	43.1
	2	16–17	213	100	1	0.5	6	2.8	5	2.3	14	6.6	38	17.8	42	19.7	107	50.2
	3	17–18	154	100	1	0.6	2	1.3	7	4.5	6	3.9	23	14.9	27	17.5	88	57.1

Table 2 shows the mean number of steps per day and 95 % CI, stratified by sex and grade level. The highest mean number of steps per day (11,659) was found among first-grade students in primary school (6–7 years old) and consistently decreased with age. The lowest mean number of steps per day (7887) was observed in students in the last year of junior high school (14–15 years old). Beyond junior high school, the mean step count modestly increased to 8485 steps per day in the first year of high school (15–16 years old), but declined as students advanced through high school (8032 steps per day at 17–18 years old). For boys, the mean number of steps per day increased from 12,575 in the first grade of primary school, peaked at 12,736 in the third grade of primary school, and subsequently ranged from 8337 to 10,218 in junior high school and from 7935 to 8583 in high school. For girls, the mean number of steps per day followed the same trend seen for both sexes combined: the highest mean number of steps per day was observed in the first grade of primary school (10,694) and consistently declined through junior high school (7437–9104), with a slight increase in the minimum value of the range in high school (8025–8398).

**Table 2:** Mean number of steps per day (with 95 % CI) among boys and girls by grade level in Tokyo in 2011

			Total			Boys			Girls			Differences of steps per day between boys and girls		
	Grade	Age	*n*	Mean steps per day	95 % CI	*n*	Mean steps per day	95 % CI	*n*	Mean steps per day	95 % CI		*p* value	d
All grades			15471	10338	10281–10395	7786	11262	11173–11351	7685	9402	9336–9468	1860	<0.001	0.513
Primary school	1	6–7	1564	11659	11504–11813	802	12575	12369–12781	762	10694	10482–10906	1881	<0.001	0.602
	2	7–8	1548	11641	11475–11806	772	12714	12491–12936	776	10573	10352–10795	2141	<0.001	0.644
	3	8–9	1565	11573	11406–11740	768	12736	12512–12961	797	10452	10232–10673	2284	<0.001	0.677
	4	9–10	1615	11336	11169–11503	828	12596	12381–12811	787	10010	9790–10231	2586	<0.001	0.758
	5	10–11	1591	10908	10740–11077	795	12302	12085–12519	796	9517	9300–9734	2785	<0.001	0.815
	6	11–12	1656	10612	10444–10781	853	12021	11807–12234	803	9117	8896–9337	2904	<0.001	0.831
Junior high school	1	12–13	1715	9674	9497–9850	877	10218	9974–10462	838	9104	8855–9353	1114	<0.001	0.299
	2	13–14	1529	9273	9092–9455	783	9830	9580–10080	746	8689	8433–8946	1141	<0.001	0.316
	3	14–15	1632	7887	7743–8031	816	8337	8136–8538	816	7437	7236–7638	900	<0.001	0.304
High school	1	15–16	374	8485	8147–8824	177	8583	8091–9075	197	8398	7932–8864	185	0.592	
	2	16–17	374	8152	7828–8477	161	8322	7827–8816	213	8025	7595–8454	297	0.373	
	3	17–18	308	8032	7692–8373	154	7935	7452–8417	154	8130	7647–8612	−195	0.574	
(subclassification)														
Primary school	1–3	6–9	4677	11624	11530–11718	2342	12674	12542–12806	2335	10571	10439–10703	2103	<0.001	0.642
	4–6	9–12	4862	10950	10853–11047	2476	12303	12175–12432	2386	9545	9414–9676	2758	<0.001	0.798
	1–6	6–12	9539	11280	11212–11348	4818	12483	12382–12584	4721	10053	9977–10129	2430	<0.001	0.718
Junior high school	1–3	12–15	4876	8950	8851–9049	2476	9476	9347–9604	2400	8408	8278–8539	1068	<0.001	0.302
High school	1–3	15–18	1056	8235	8042–8428	492	8294	8007–8582	564	8184	7915–8452	110	0.575	

Differences between boys and girls tested with independent t-tests and Cohen’s *d*s were calculated to assess the size of intergroup differences

During primary and junior high school, the step count was significantly higher among boys by 2000 and 1000 steps per day, respectively, compared with girls at the same grade level. However, during the high school years, there was no significant sex difference in numbers of daily steps (*p* = 0.592 for high school level 1 [15–16 years], *p* = 0.373 for level 2 [16–17 years], and *p* = 0.574 for level 3 [17–18 years]), with boys taking an absolute average of only 200 steps per day more than girls (Table 2). Table 3 shows the minimum, maximum, and percentile values for the number of steps per day in each school grade, stratified by sex.

**Table 3:** Normative steps per day by grade level and sex in Tokyo in 2011

				Percentiles																							
	Grade	Age	Minimum	1	3	5	10	15	20	25	30	35	40	45	50	55	60	65	70	75	80	85	90	95	97	99	Maximum
Boys																											
Primary school	1	6–7	1523	2767	6569	7563	8743	9310	9996	10423	10742	11259	11663	12010	12397	12905	13292	13776	14146	14794	15268	16021	16813	18004	18886	22157	24315
	2	7–8	1005	4789	6582	7264	8509	9210	9822	10314	10822	11205	11732	12268	12660	13108	13572	14002	14336	14860	15408	16305	17101	18630	19725	22100	26775
	3	8–9	1032	4639	6148	7183	8377	9467	9939	10345	10880	11293	11763	12128	12563	13059	13483	13983	14496	14962	15522	16264	17187	18627	19893	21601	29999
	4	9–10	1591	4343	6105	6971	7937	8929	9710	10260	10680	11027	11579	12087	12536	13027	13491	13927	14506	14952	15606	16284	17344	18436	19545	21291	27000
	5	10–11	1987	4340	6136	7091	8019	8774	9345	9760	10236	10613	11229	11556	11992	12460	12864	13391	13885	14478	15087	16174	17083	18567	19522	21678	29994
	6	11–12	1846	3967	5609	6321	7298	8309	8910	9546	9991	10508	10908	11389	11726	12168	12794	13248	13825	14355	14977	15675	16684	18540	19457	21787	30000
Junior high school	1	12–13	1035	2535	4290	4785	5892	6454	7104	7591	7929	8294	8731	9122	9516	9982	10605	11198	11811	12441	13186	14250	15430	17732	18775	21286	29265
	2	13–14	1034	2544	3822	4252	5304	6169	6644	7120	7524	8013	8362	8735	9224	9645	10242	10755	11472	12173	12832	13727	15003	17200	18263	21536	27659
	3	14–15	1000	2096	3324	3882	4800	5283	5789	6069	6539	6870	7135	7477	7802	8160	8642	8994	9428	9973	10441	11249	12381	14799	16217	19984	26603
High school	1	15–16	2171	3235	3922	4214	4719	5298	5592	6212	6560	6951	7268	7488	7951	8187	8578	9009	9516	10012	10641	11556	13316	16905	18602	21309	24046
	2	16–17	1410	2108	3016	3288	4185	4606	5246	5714	6079	6695	6988	7408	7760	8158	8560	8764	8984	9662	10952	12146	14043	16059	17676	20211	20311
	3	17–18	1007	1146	2922	3317	3914	4372	5033	5343	6118	6393	6733	7081	7439	7758	8519	9114	9354	10007	10375	11354	12366	14254	15746	18292	19257
Girls																											
Primary school	1	6–7	1269	3846	6201	6807	7668	8172	8621	8939	9262	9647	10072	10424	10715	10972	11263	11585	11886	12293	12746	13251	13837	14779	15708	17307	21908
	2	7–8	1511	3987	5933	6563	7352	7806	8346	8737	9128	9415	9767	10114	10441	10766	11148	11492	11824	12318	12712	13295	13966	15249	16348	17919	20879
	3	8–9	1315	4018	5698	6197	7224	7818	8269	8668	9028	9327	9645	9935	10219	10639	11025	11299	11625	12095	12557	13323	14019	14954	15796	17877	24133
	4	9–10	1549	4121	5544	6131	6789	7297	7877	8190	8551	8960	9315	9716	9952	10176	10450	10772	11101	11469	11943	12482	13186	14596	15476	17789	22450
	5	10–11	2697	4024	5110	5637	6461	7020	7508	7878	8163	8492	8732	9099	9372	9698	10019	10317	10669	10977	11415	11887	12777	13745	14944	15826	22195
	6	11–12	1953	3953	5171	5590	6400	6791	7067	7487	7753	8033	8252	8622	8884	9234	9532	9832	10103	10427	10902	11399	12153	13626	14504	16596	20504
Junior high school	1	12–13	1833	2880	3777	4445	5231	5758	6296	6649	7001	7386	7766	8265	8648	8969	9410	9879	10426	11077	11856	12760	14001	15518	16216	18181	23343
	2	13–14	1451	3176	4042	4466	5298	5786	6231	6572	6909	7211	7521	7873	8212	8542	8820	9273	9732	10151	10791	11519	12893	15101	16404	18749	24388
	3	14–15	1346	2705	3640	4213	4711	5129	5447	5757	6134	6374	6669	6862	7144	7386	7699	8089	8408	8707	9140	9806	10474	11687	12625	14656	20730
High school	1	15–16	1086	2037	3362	3797	4722	5103	5828	6369	7027	7277	7530	7798	8141	8403	8955	9329	9689	9971	10623	11447	12799	14323	14889	16004	17258
	2	16–17	3126	3227	3814	4396	5091	5191	5628	6108	6454	6903	7142	7388	7656	8010	8351	8663	9024	9320	9919	10570	11422	12787	14899	16251	20525
	3	17–18	3000	3115	3646	4518	4966	5565	6135	6292	6684	6942	7158	7462	7592	7864	8320	8697	9305	9694	9989	10531	11570	14000	14864	16570	17308

Table 4 compares the results of this study, which used Yamasa EX-200 pedometers, and those of the CANPLAY survey, which used Yamax Digiwalker SW-200 pedometers [16]. The mean number of daily steps taken by boys in primary school was similar for the two surveys. However, at the higher-grade levels, pedometer-determined PA levels were lower for boys living in Tokyo than for their Canadian counterparts by approximately 1000 to 2000 steps per day. Similarly, the number of steps taken per day by girls living in Tokyo was lower by approximately 1000 steps per day than that of Canadian girls across all grades.

**Table 4:** Comparison of mean number of steps per day among boys and girls between Canada (in 2005–2011) and Tokyo in 2011

	Boys		Girls	
	Canada^a^	Tokyo	Canada^a^	Tokyo
Pedometer	Yamax SW-200	Yamasa Ex-200	Yamax SW-200	Yamasa Ex-200
Age	Steps per day	Steps per day	Steps per day	Steps per day
6	12,435	12,575	11,627	10,694
7	12,700	12,714	11,507	10,573
8	12,989	12,736	11,435	10,452
9	13,097	12,596	11,490	10,010
10	13,030	12,302	11,638	9517
11	12,694	12,021	11,367	9117
12	12,211	10,218	10,510	9104
13	11,816	9830	10,122	8689
14	11,114	8337	9988	7437
15	10,650	8583	9476	8398
16	10,344	8322	9252	8025
17	10,493	7935	9343	8130

^a^The Canadian Physical Activity Levels Among Youth (CANPLAY) survey [16]. Yamax SW-200 pedometers were used in the CANPLAY survey, and Yamasa EX-200 pedometers in the Tokyo survey. The SW-200 was worn on a belt, the EX-200 was placed in a pocket

**Fig. 2:**
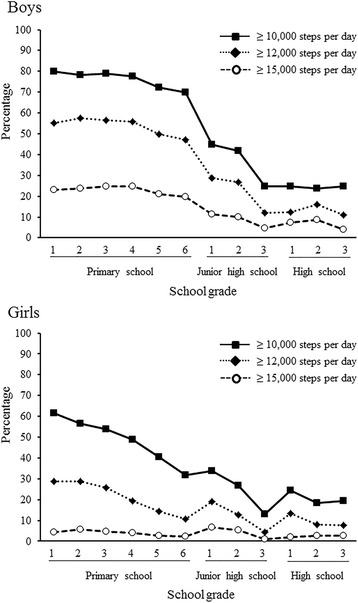
Proportion of students taking ≥10,000, ≥12,000, or ≥15,000 steps per day by sex and school grade

The proportion of students taking ≥10,000, ≥12,000, and ≥15,000 steps per day was 60.5, 41.2, and 17.4 %, respectively, for boys and 39.1, 17.6, and 3.9 %, respectively, for girls (Fig. 2). Boys showed a distinct decrease in the number of steps per day between the sixth primary school grade and the first junior high school grade. Girls showed a gradual decline in the number of steps per day from the start to the finish of primary school, with a slight increase during the first year of junior high school.

**Fig. 3:**
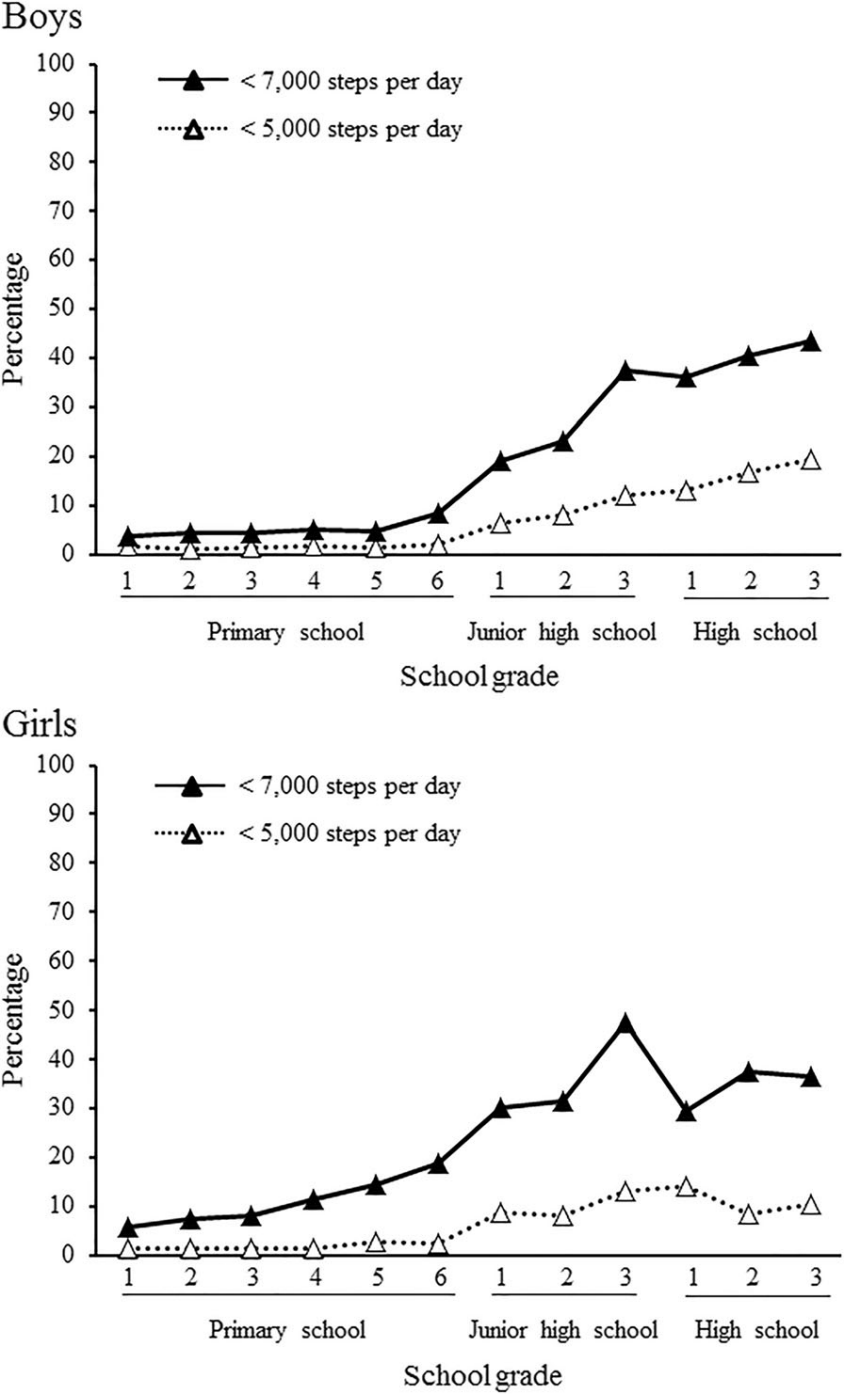
Proportion of students taking <7,000 or <5,000 steps per day by sex and school grade

The proportion of students taking <7000 and <5000 steps per day was 14.1 and 4.7 %, respectively, for boys and 20.7 and 5.0 %, respectively, for girls (Fig. 3). The proportion of boys taking <7000 and <5000 steps per day rapidly increased between the sixth grade of primary school and the first grade of junior high school, and continued to increase toward high school. The proportion of girls taking <7000 steps per day gradually increased from the first grade level to the last year of junior high school. There was a moderate relative decrease in the proportion of girls taking <7000 steps per day in the first year of high school, but a subsequent steady increase in later years.

## Discussion

Using a representative sample of the Tokyo metropolitan area, this is one of the largest surveys worldwide to investigate pedometer-determined PA levels in children and adolescents. The results indicate that in primary school (age 6–12 years), junior high (age 12–15 years), and high school (age 15–18 years), boys took an average of 12,483, 9476, and 8294 steps per day, respectively, while girls took an average of 10,053, 8408, and 8184 steps per day, respectively. The mean number of daily steps was significantly higher for boys than for girls through 6 to 15 years, with an overall decreasing age-related trend for both sexes. Boys tend to be more active than girls at most ages, although this difference disappears in high school, and a reduction in PA levels from childhood to adolescence has been previously reported [17, 18, 25]. Until now, there has been limited objective data for large-scale evaluations of PA levels in Japanese children and adolescents [5]. The step patterns of our Tokyo students are similar to those reported in a review of pedometer data from 43 studies of young people in 13 countries [30].

Although high school is not compulsory in Japan, more than 95 % of students in Tokyo attend high school after passing their entrance examination [19]. Generally, Japanese students in the third year of junior high school spend substantial time studying for this examination. Therefore, students in their final junior high school year may focus more on studying than on PA, which may explain why this group has the lowest overall mean step count and the highest proportion of youth accumulating fewer steps per day relative to the two indices of a sedentary lifestyle.

The differences in PA levels between children in Tokyo and those in Canada can be interpreted in four ways. First, Canadian and Japanese children may actually have different PA levels. Second, this difference may result from variations in the survey-specific pedometers used and their positioning. The EX-200 (used in this survey) is an in-pocket pedometer and the SW-200 (used in the CANPLAY survey) is worn on a belt. The EX-200 is a triaxial accelerometer with a filter function that monitors continuous walking activity to recognize actual steps; it is programmed to count steps when an individual takes ≥10 steps without pausing for <2 s (e.g., if a subject moves <10 steps and pauses for ≥2 s, the previous steps will not be counted). Silcott et al. [31] reported that pedometers with a filter function might underestimate step counts compared with pedometers without a filter function. Although there is no data directly comparing steps measured by the EX200 and the SW-200, Tanaka et al. reported that the EX-200 underestimated step counts by 7.9 % compared with the Kenz Lifecorder among children aged 6 to 12 years [22] (additionally, Schneider et al. reported no significant difference in step count values between the Kenz Lifecorder and the SW-200 [21]). These findings suggest that the EX-200 step counts may be lower than those obtained by the SW-200. Thus, differences among devices should be considered. Third, the sampling method in this TMBE-administered survey was different from the CANPLAY which employed random sampling and collected data through the mail. In contrast, the Tokyo survey asked each municipality to choose one primary and one junior high school from its district. All geographical areas throughout Tokyo were covered by this method. However, it is uncertain whether this sampling method lead to underestimation or overestimation of the step counts. Finally, children’s activity levels may be affected by seasonal variation [32]. Craig et al. [15] reported that Canadian children’s PA is lower in the fall and winter than in the spring and summer. Because this survey was conducted in the fall in Tokyo, further study is needed to determine the effect of seasonal changes on PA levels in Japanese children and adolescents.

Vincent et al. [33] assessed pedometer-determined daily step counts in a convenience sample of children aged 6 to 12 years in the U.S. (*n* = 711), Sweden (*n* = 680), and Australia (*n* = 563). Although there are potential issues with the sample size, sampling bias, and the different pedometers used in the present survey, the Tokyo survey reported approximately 3000 and 1000 fewer steps for boys per day than Swedish and Australian boys, respectively, but a similar level of activity to American boys. We observed a similar pattern for girls, who took approximately 2000 and 1000 fewer steps per day than Swedish and Australian girls, respectively, but accumulated a similar mean number of steps per day as American girls.

There are no clear guideline recommendations for number of steps per day for children and adolescents. However, we interpreted the present data in the context of previously published step-defined criteria. Certainly, it is optimal to use criteria that have been established based on health-related values. However, practical effectiveness should also be taken into consideration when setting any single criterion. For example, if almost all (or very few) people meet a specified value, then its practical effectiveness for educational and public health purposes is questionable. The present results include implications regarding the practical effectiveness of various criteria. We found that the proportion of boys and girls meeting specific criteria (i.e., 10,000, 12,000, and 15,000 steps per day) decreased with age. Additionally, distinct sex-specific patterns were observed. The Tokyo Metropolitan Government has recommended ≥15,000 steps per day for children and adolescents, regardless of age or sex [27]. However, our findings reveal that many children and adolescents (except primary school boys) do not meet this target. Therefore, a criterion of ≥15,000 steps per day seems very high and thus not practically effective, especially for girls. Two courses of action might improve the situation. First, appropriate age- and sex-specific targets may be set. Second, a graduated scale of values might describe PA distribution better than a single target value and may encourage less active children to improve their PA level. Although it seems too low as an optimal value for health, a criterion of ≥10,000 steps per day showed dynamic patterns across age and sex in this survey. This suggests that ≥10,000 steps per day is a practically effective criterion for evaluating lifestyle changes/differences across age and sex for education and public health purposes. For children, <7000 steps per day has been suggested as an appropriate sedentary lifestyle index [15, 28]. In this survey, the proportion of students meeting this criterion increased with age as anticipated. However, its practical effectiveness was limited for primary school boys, who accumulated a higher average number of steps per day than this value, suggesting the need for age- and sex-specific values on a graduated scale for youth.

In the present study, the use of unsealed pedometers meant that participants were aware of their step counts, potentially leading to reactivity bias. Because TMBE did not record the steps per day for the first 7 days, our ability to test for reactivity was hampered. However, Craig et al. [23] showed no evidence of reactivity in a population sample of 5- to 19-year-olds wearing unsealed pedometers for 7 days. Other studies have reported no evidence of reactivity bias and have generally concluded that this is not a problem when evaluating children [13]. Additionally, Clemes and Deans [34] reported that the reactivity effect diminishes after the first week of monitoring, returning to normal levels in the second week. Therefore, the data obtained in the second week of our 2-week surveillance were probably not systematically affected by reactivity bias.

### Study strengths and limitations

This study has several strengths. Because the participants were sampled throughout Tokyo, their mean number of steps per day is representative of PA levels in Tokyo youth. Using the same adjusted treatment methods for pedometer data as used in the CANPLAY study enabled between-study comparisons. Finally, in this study, more than 86 and 90 % of boys and girls, respectively, wore their pedometers for a minimum of 4 days; it has been reported that 4 or more valid days of data in youth enhances data reliability [35].

Study limitations must be acknowledged. First, this was that was a secondary analysis of a survey conducted by an education authority and we had no input regarding the original study design. Despite this, the survey represents an important source of objectively monitored data on children. Although this is the largest study of in-pocket pedometer-determined PA in youth (and is thus a useful reference data source for others using this type of device), these pedometers do tend to underestimate absolute step-defined PA levels. Regardless, it is reasonable to assume that the observed data trends are valid. Second, the lack of private school students in the sample may influence the results. In 2011, the proportion of students in the Tokyo metropolitan area attending private primary, junior high, and high schools was 4.5, 25.5, and 55.9 %, respectively [36]. The difference in tuition costs for private schools may indicate differences in familial socioeconomic status. If socioeconomic status affects youth PA levels, the present data may not accurately reflect PA in the larger Tokyo youth population. Third, the TMBE survey complied with the organization’s safety policy, thus allowing students to remove their pedometers during vigorous full-contact activity (unfortunately, this was not tracked); this may have underestimated the overall number of steps per day. Finally, the issue of wearing compliance should be considered. Students recorded their step counts at school under the guidance of trained teachers. However, no other methods were employed to confirm whether they actually wore the pedometers as directed.

## Conclusion

This study demonstrates that children’s pedometer-determined PA generally decreases with age and that there is a substantial difference in the number of steps taken per day between boys and girls in Tokyo. The PA decrease was greater in boys because they achieved initial higher peak values; once the students reached high school, the sex difference in the number of steps per day disappeared. These findings contribute to our current understanding of the PA levels of youth living in Tokyo and will be useful for surveillance, screening, and comparison purposes, as well as planning strategies.

## Abbreviations

CANPLAY: Canadian physical activity levels among youth
CI: Confidence interval
PA: Physical activity
TMBE: Tokyo Metropolitan board of education
